# Citrulline-enhanced chicken resistance to *Salmonella Enteritidis* infection via urea cycle modulation and nitric oxide production

**DOI:** 10.1128/spectrum.01546-25

**Published:** 2025-11-11

**Authors:** Ming Jiang, Pei-pei Hu, Wei-min Li, Jie Ma, Wei Luo, Man-shan Cai, Chu-xiao Lin, Chun-lin Xie, Jian-min Zhang, Jian Ji

**Affiliations:** 1 State Key Laboratory of Swine and Poultry Breeding Industry, Guangdong Key Laboratory of Animal Breeding and Nutrition, Institute of Animal Science, Guangdong Academy of Agricultural Sciences 656960 , Guangzhou, China; 2 College of Life Sciences, Hunan Normal University 554899, Changsha, China; 3 College of Veterinary medicine, South China Agricultural University 554665, Guangzhou, China; Institut National de Santé Publique du Québec, Sainte-Anne-de-Bellevue, Québec, Canada

**Keywords:** *Salmonella Enteritidis*, chicken, citrulline, nitric oxide, metabolomics

## Abstract

*Salmonella Enteritidis* infection has caused substantial economic losses in the poultry industry. Traditional antibiotic treatments have led to issues, such as drug-resistant bacteria and antibiotic residues, which pose significant threats to human and animal health, as well as food and environmental safety. Therefore, establishing new, effective prevention and control methods is of paramount importance. In this study, we utilized gas chromatography-mass spectrometry (GC-MS) metabolomics to analyze the metabolomic profiles of chickens infected with varying concentrations of *Salmonella Enteritidis*. Significant differences in the metabolomes were observed, with citrulline identified as a candidate biomarker, showing increased levels corresponding to higher infection doses. Exogenous addition of citrulline was found to upregulate the expression of the inducible nitric oxide synthase (iNOS) gene in the urea cycle of chicken HD11 macrophages, leading to increased nitric oxide (NO) levels and enhanced phagocytosis of *Salmonella Enteritidis* by HD11 cells. Furthermore, *in vivo* challenge experiments demonstrated that exogenous citrulline improved the ability of chickens to clear *Salmonella Enteritidis* and increased their survival rate post-infection. Importantly, this effect could be attenuated by the iNOS inhibitor 1400W. Thus, our study presents a novel strategy for combating bacterial infections through metabolic modulation.

**IMPORTANCE:**

Chickens respond to *Salmonella* infection by adjusting the metabolic state of their bodies. Citrulline can enhance the phagocytic ability of phagocytes by strengthening the urea cycle. *In vitro* clinical trials have revealed that citrulline can increase the survival rate of chickens after *Salmonella* infection.

## INTRODUCTION

*Salmonella Enteritidis* is a non-spore-forming, non-capsulated, Gram-negative enteric bacillus widely distributed in nature. This bacterium not only causes illness and death in poultry, leading to significant economic losses, but also infects humans through poultry, eggs, milk, dairy products, and other animal products, primarily eggs and poultry meat (1, 2). These infections result in food poisoning and can even be fatal. In Europe, the economic impact of *Salmonella Enteritidis* due to contaminated poultry eggs amounts to an estimated 3 billion EUR/year (3). *Salmonella Enteritidis* induces acute gastroenteritis (food poisoning) in humans, with an increasing incidence globally, making it a critical international public health concern. Poultry is identified as the principal source of *Salmonella Enteritidis* (4, 5). Currently, in the poultry industry, *Salmonella Enteritidis* is managed with antibiotics. However, prolonged antibiotic use poses public health risks, such as drug residues and the emergence of antibiotic-resistant strains, including the potential development of superbugs. Antibiotic resistance is a longstanding global medical challenge and has been identified by the World Health Organization as one of the major public health threats of the 21st century (6, 7). Consequently, there is an urgent need to develop effective new strategies for the prevention and control of *Salmonella Enteritidis* in poultry.

Enhancing the innate immune response of poultry represents an effective strategy for combating infections. Innate immunity serves as the primary defense mechanism against microbial infections in hosts. Within this defense system, host cells utilize various pattern recognition receptors to detect bacterial components, triggering a cascade of molecular reactions that enhance the host’s capacity to eliminate bacteria through the activation of inflammatory cytokines (8). Therefore, enhancing host immunity presents a promising approach for combating bacterial infections. Recent studies have demonstrated that the efficacy of innate immune responses against bacterial infections is influenced by the host’s metabolic status (9–12). Our early investigations have yielded some findings: (i) we have revealed that inosine and succinate regulate innate cellular and humoral immunity. These metabolites competitively bind to proline hydroxylase, impacting the stability of hypoxia-inducible factor and thereby modulating the intensity of immune responses. Exogenous administration of inosine has been shown to enhance the survival rates of septic mice (13). (ii) We also have linked alanine metabolism intensity to resistance against *Vibrio parahaemolyticus* in mice. Supplementation of alanine enhances bacterial clearance and improves survival post-infection. Mechanistic studies indicate that alanine regulates TLR4 expression and dimerization through metabolic reprogramming, generating palmitic acid to activate downstream immune response pathways against infections (14). (iii) Additionally, we have also demonstrated that maltose increases host lysozyme gene expression and enhances lysozyme binding efficiency to resistant bacteria, thereby augmenting bacterial clearance capabilities (15). Based on these findings, it is hypothesized that modifying the metabolic state of poultry to enhance immune responses could provide a viable strategy for preventing and treating *Salmonella Enteritidis* infections without relying on antibiotics.

This study employed varying doses of *Salmonella Enteritidis* to challenge chickens and utilized gas chromatography-mass spectrometry (GC-MS) metabolomics to analyze spleen samples post-infection. Bioinformatics analysis revealed that upregulation of arginine metabolism is a significant response to infection, and citrulline was identified as a candidate biomarker. *In vivo* experiments have demonstrated that citrulline enhances the ability of chickens to clear *Salmonella Enteritidis* and improves their survival rate following infection.

## RESULTS

### The phenotypes of chickens infected by *Salmonella Enteritidis*

**Fig 1:**
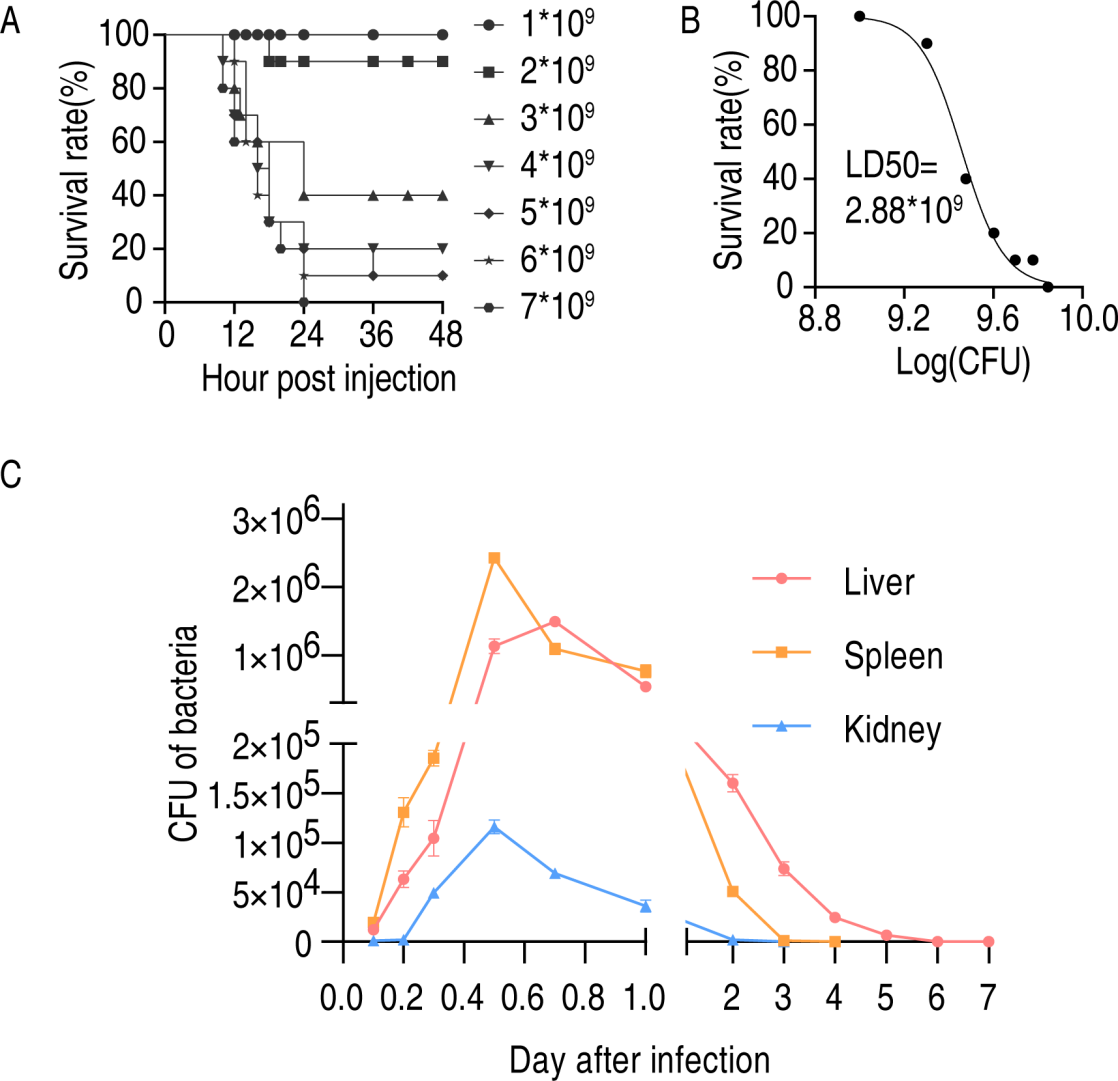
The survival rates and bacteria loads of *Salmonella Enteritidis* challenged chicken. (**A**) The survival rates after different concentrations of bacterial challenge infections. (**B**) The half-lethal dose (LD50) was calculated by GraphPad Prism 9.0 using the data in panel **A**. (**C**) Liver, spleen, and kidney bacteria loads at different days after infection by 1 × 10^8^ CFU *Salmonella Enteritidis*.

The strain used for the challenge in this experiment was *Salmonella Enteritidis* (no.: CMCC50041). After the bacteria were retrieved from the −80°C freezer (1 mL total volume), they were rapidly thawed and transferred to 100 mL of LB medium for culture. Once the OD600 reached 1.0, the bacteria were harvested, washed with saline, and subsequently administered via intraperitoneal injection. To determine the median lethal dose (LD50) of this strain in chickens, seven bacterial concentrations were used for the challenge, ranging from 1 to 7 × 10^9^ CFU per chicken (Fig. 1A). After a 3-day observation period, mortality rates were recorded, and the data from Fig. 1A were analyzed using GraphPad software, resulting in a calculated LD50 of 2.88 × 10^9^ CFU (Fig. 1B). Subsequently, we evaluated the clearance of *Salmonella Enteritidis* in chickens. The challenge dose was a sublethal dose of 1 × 10^9^ CFU per bird. Spleen, liver, and kidney samples were collected at various time points post-challenge for bacterial enumeration. The results indicated peak bacterial loads in the spleen, liver, and kidneys at 0.5, 0.7, and 0.5 days, respectively, with complete clearance observed after 7, 4, and 3 days, respectively (Fig. 1C).

### Metabolite profiles of chickens exposed to *Salmonella Enteritidis*

**Fig 2:**
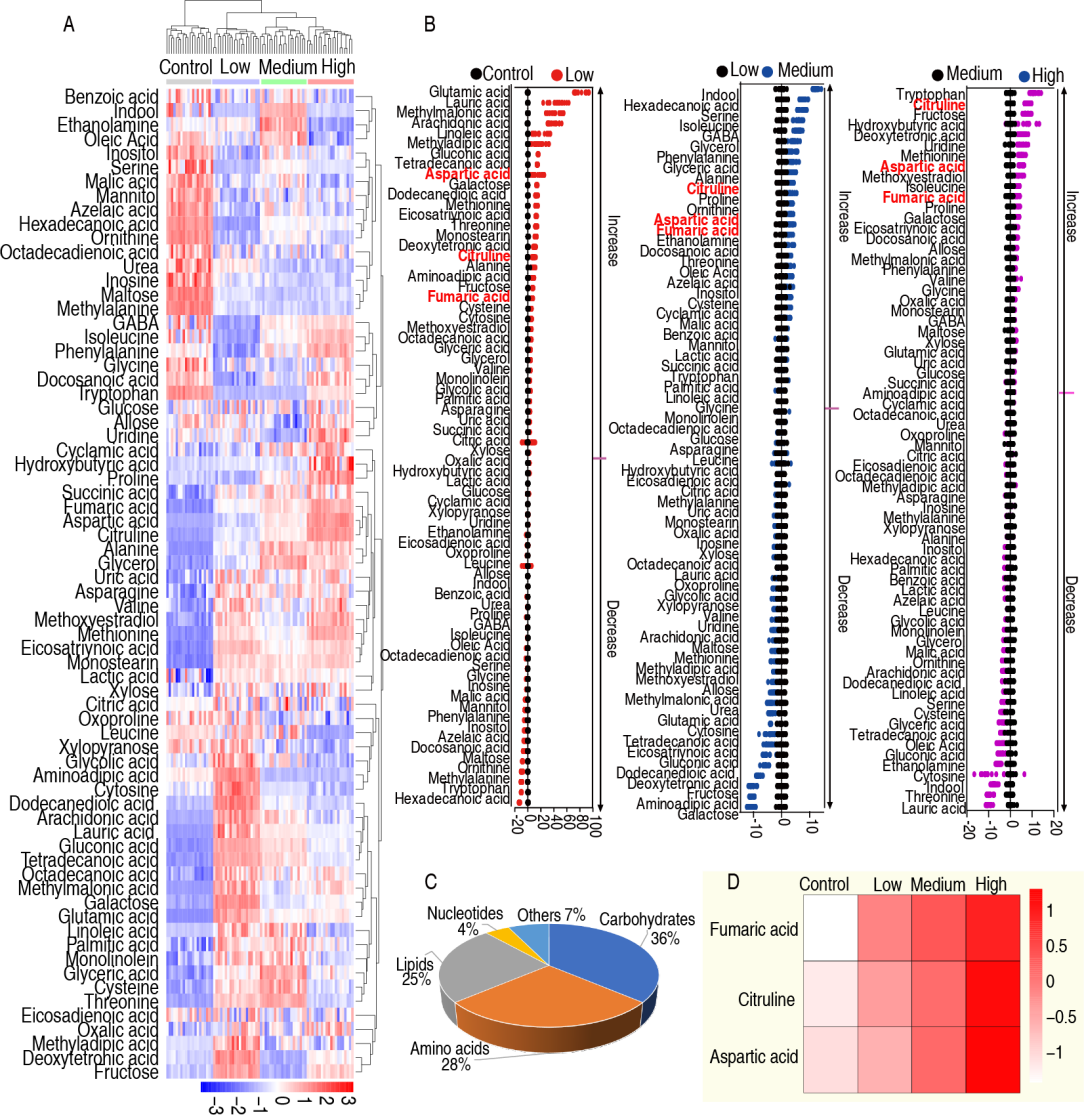
Metabolomics analysis of chicken infected with low, medium, and high dose *Salmonella Enteritidis.* (**A**) Heatmap of chicken spleen metabolomics infected with 1 × 10^7^ (low dose), 1 × 10^8^ (medium dose), or 1 × 10^9^ CFU (high dose) *Salmonella Enteritidis*. Red and blue indicate the increase and decrease of the metabolites scaled to the mean and standard deviation of the row metabolite level, respectively. (**B**) Z scores (standard deviation from the average) of low, medium, and high dose infection groups with the control group. (**C**) The categories of the metabolites. (**D**) Metabolites that increased with the challenged dose.

Innate immunity plays a crucial role in the host’s defense against bacterial infections, with the spleen being a primary site for the production of innate immune cells. The spleen has been extensively studied in the context of innate immunity across various animal models, including mice, zebrafish, and chickens (13–16). In this experiment, bacterial challenges were conducted using 1 × 10^7^ (low), 1 × 10^8^ (medium), and 1 × 10^9^ (high) CFU/chicken. The control group received an equivalent dose of normal saline. Each group consisted of 10 chickens. Six hours post-challenge, spleen samples were collected for metabolomic analysis. Each sample was analyzed in duplicate, resulting in a total of 80 data sets. The metabolomic data were analyzed, identifying 72 differential metabolites, which were visualized using a heat map (Fig. 2A). These metabolites were further categorized, revealing that carbohydrates accounted for 36%, amino acids for 28%, fatty acids for 25%, nucleic acids for 4%, and other substances for 7% (Fig. 2C). To illustrate the variations in metabolite levels between different doses and between the challenge and control groups, Z-scores were calculated. The results indicated that the levels of fumaric acid, citrulline, and aspartic acid increased progressively with higher challenge doses (Fig. 2B and D).

### Biomarker identification

**Fig 3:**
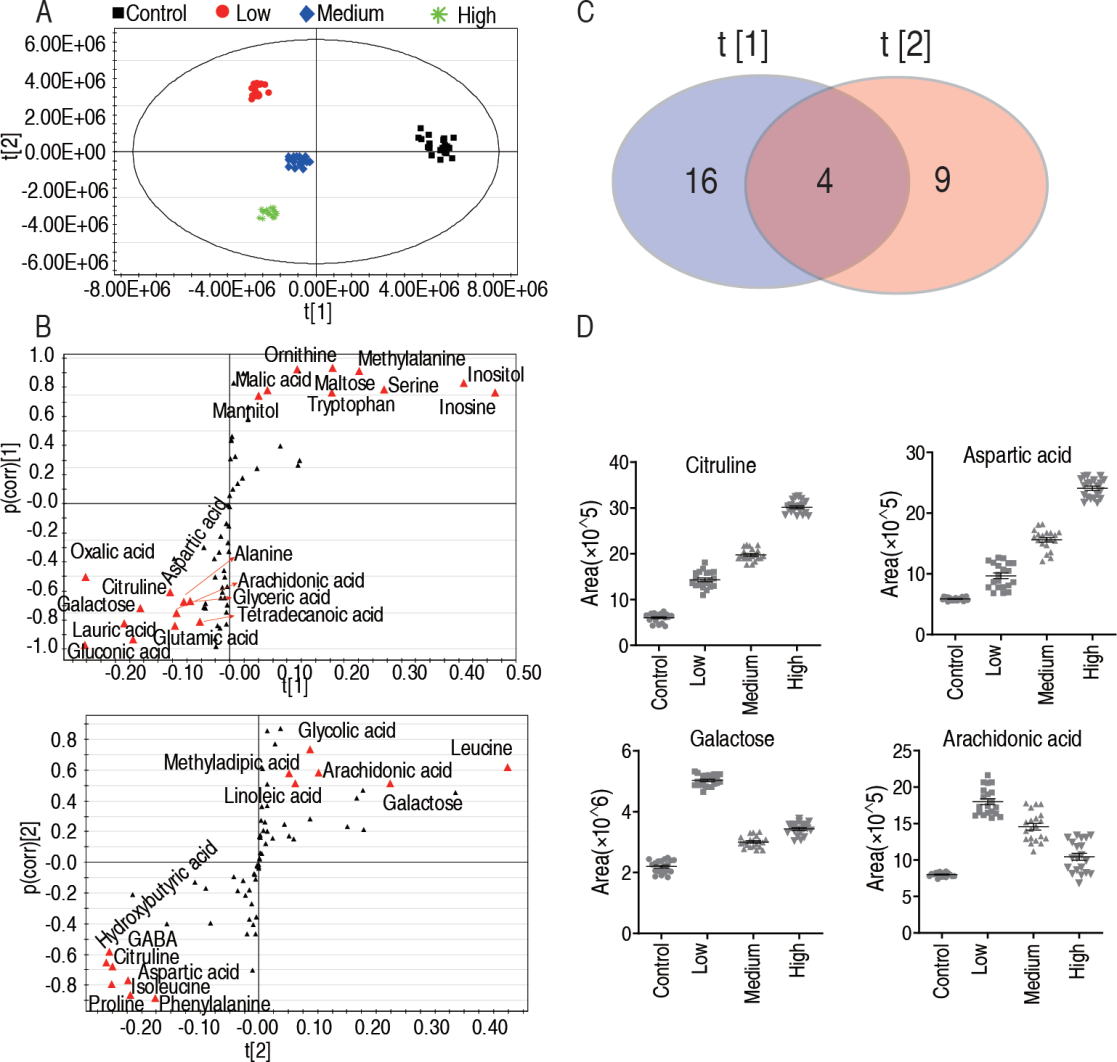
Target prediction analysis of candidate metabolite. (**A**) Principal-component analysis. Each dot represents one technical replicate. (**B**) The distribution of differential abundance of metabolite weight from PLS-DA to samples. Triangles represent metabolites. Potential biomarkers are highlighted in red. (**C**) Wayne diagram of biomarkers in factor t [1] and t [2] in panel B. (**D**) The overlapping metabolites in Wayne diagram.

The metabolomics data from the above determinations were analyzed using principal component analysis (PCA) and orthogonal partial least squares discriminant analysis (OPLS-DA) with SIMCA-P 12.0 software. The PCA results indicated that factor t [1] could differentiate between the control group and the infection group, while factor t [2] could distinguish among the low-dose, medium-dose, and high-dose groups (Fig. 3A). The OPLS-DA analysis revealed that factor t [1] identified 20 target substances, specifically inositol, inosine, serine, methylalanine, maltose, tryptophan, ornithine, malic acid, mannitol, oxalic acid, aspartic acid, citrulline, alanine, arachidonic acid, glyceric acid, tetradecanoic acid, glutamic acid, lauric acid, gluconic acid, and galactose. Factor t [2] identified 13 target substances, including leucine, glycolic acid, arachidonic acid, methyladipic acid, linoleic acid, hydroxybutyric acid, GABA, citrulline, aspartic acid, isoleucine, proline, and phenylalanine (Fig. 3B). A Venn diagram of the candidate target substances from factors t [1] and t [2] showed four common metabolites: citrulline, aspartic acid, galactose, and arachidonic acid (Fig. 3C). A scatter plot was then constructed to illustrate the abundance of these four metabolites (Fig. 3D).

### Pathway analysis of differential metabolites

**Fig 4:**
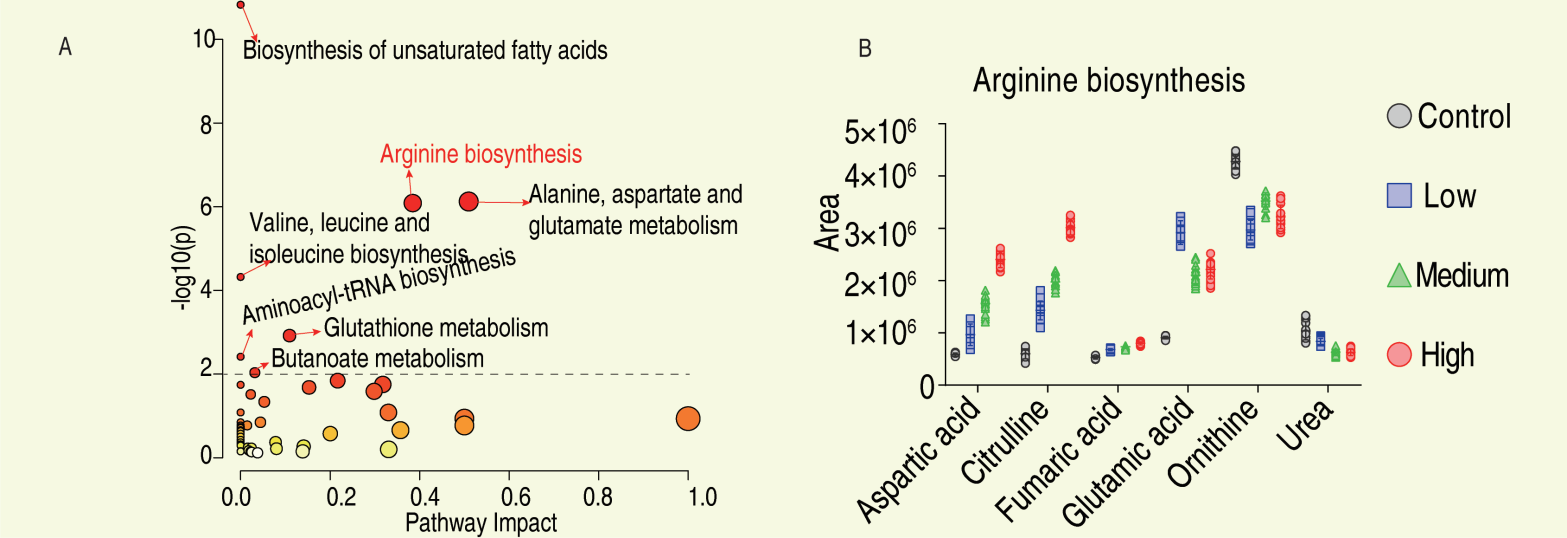
Pathways analysis of the metabolites. (**A**) The enriched pathway of the differential metabolites. Significantly enriched pathways were annotated (−log10(p) ≥ 2). (**B**) Abundance of metabolites of arginine biosynthesis.

To identify the metabolic pathways involved in the differential metabolites, pathway enrichment analysis was performed using MetaboAnalyst 5.0 (https://www.metaboanalyst.ca/). Using a threshold of −log2(p) ≥ 2, seven metabolic pathways were identified, ranked by their impact value: alanine, aspartate, and glutamate metabolism; arginine biosynthesis; glutathione metabolism; butanoate metabolism; aminoacyl-tRNA biosynthesis; valine, leucine, and isoleucine biosynthesis; and biosynthesis of unsaturated fatty acids (Fig. 4A), with arginine biosynthesis ranking second. To visually depict the trends in metabolic pathways, scatter plots showing changes in metabolite content within each pathway were created (Fig. 4B; Fig. S1). Integrating the results of the candidate target substances from Fig. 2D and 3D, it was observed that aspartic acid and citrulline are common substances, both of which belong to the arginine biosynthesis pathway. Therefore, it is speculated that arginine metabolism may play a role in the process of chicken resistance to *Salmonella Enteritidis* infection.

### Enhanced nitric oxide content during *Salmonella Enteritidis* infection

**Fig 5:**
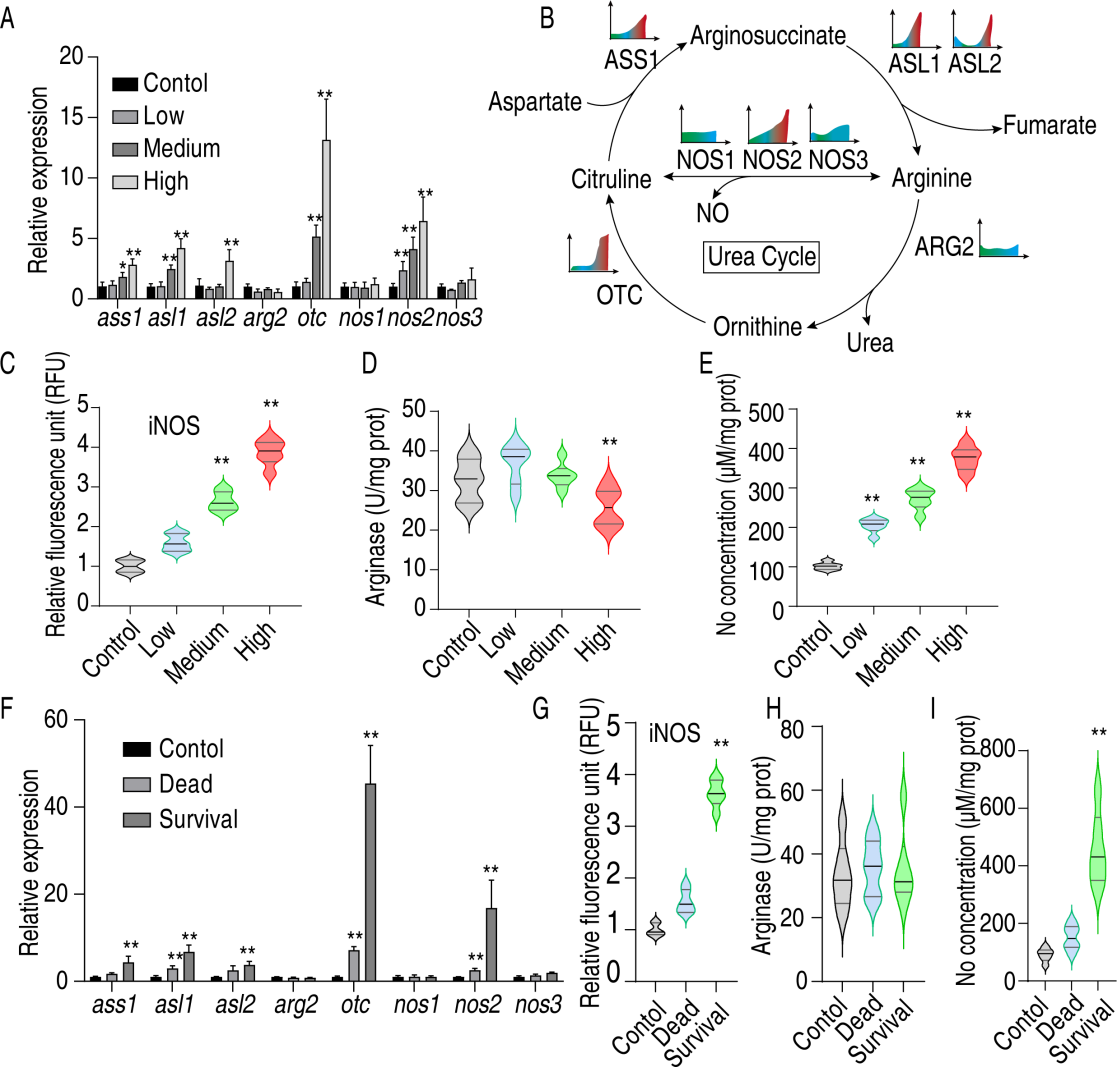
Effect of *Salmonella Enteritidis* infection on arginine biosynthesis of chicken in *vivo*. (**A**) Transcript levels of eight urea cycle-related genes against 1 × 10^7^ (low dose), 1 × 10^8^ (medium dose), or 1 × 10^9^ CFU (high dose) *Salmonella Enteritidis* infection. (**B**) Schematic diagram of the urea cycle and the color scale of gene expression in panel A. (**C**) Inducible nitric oxide synthase (iNOS) activity, (**D**) arginase activity, (**E**) NO concentration level in chicken following low, medium, and high dose *Salmonella Enteritidis* infection. (**F**) Transcript levels of eight urea cycle-related genes in survival or dying chicken following 3 × 10^9^ CFU *Salmonella Enteritidis* infection. (**G**) iNOS activity, (**H**) arginase activity, (**I**) NO concentration level in survival or dying chicken following 3 × 10^9^ CFU *Salmonella Enteritidis* infection. **P < 0.01.

The urea cycle is a crucial component of the arginine biosynthesis pathway and plays a role in the host’s innate immune response (17–19). Therefore, the expression of urea cycle-related genes was analyzed using fluorescence quantitative PCR, including eight genes: *ass1, asl1, asl2, arg2, otc, nos1, nos2, and nos3*. The results indicated that the expression of *ass1, asl1, asl2, otc, and nos3* increased with the challenge dose (Fig. 5A). To visually represent gene expression, a heat map was generated, where higher expression levels are indicated by redder colors (Fig. 5B). The data showed that the ornithine-citrulline-arginine-NO pathway is activated post-infection, whereas the arginine-ornithine pathway remains silent.

To further validate these findings, the enzyme activity of inducible nitric oxide synthase (iNOS) and arginase, as well as nitric oxide (NO) content, was measured. The results demonstrated that iNOS activity and NO content increased with the infection concentration, while arginase activity was inhibited at higher concentrations (Fig. 5C through E). Additionally, chickens were challenged with a lethal dose of 3 × 10^9^ CFU, and spleen samples were collected from both dying and surviving chickens. The analysis revealed that the ornithine-citrulline-arginine-NO pathway was activated in the surviving group, whereas the arginine-ornithine pathway was inactive (Fig. 5F). Enzyme activity and NO content tests confirmed that NO levels and iNOS activity were higher in the surviving group compared to the dying group (Fig. 5G through I). These findings suggest that enhancing NO production can improve chickens’ resistance to *Salmonella Enteritidis* infection.

### Elevated phagocytic ability of HD11 cells by exogenous citrulline

**Fig 6:**
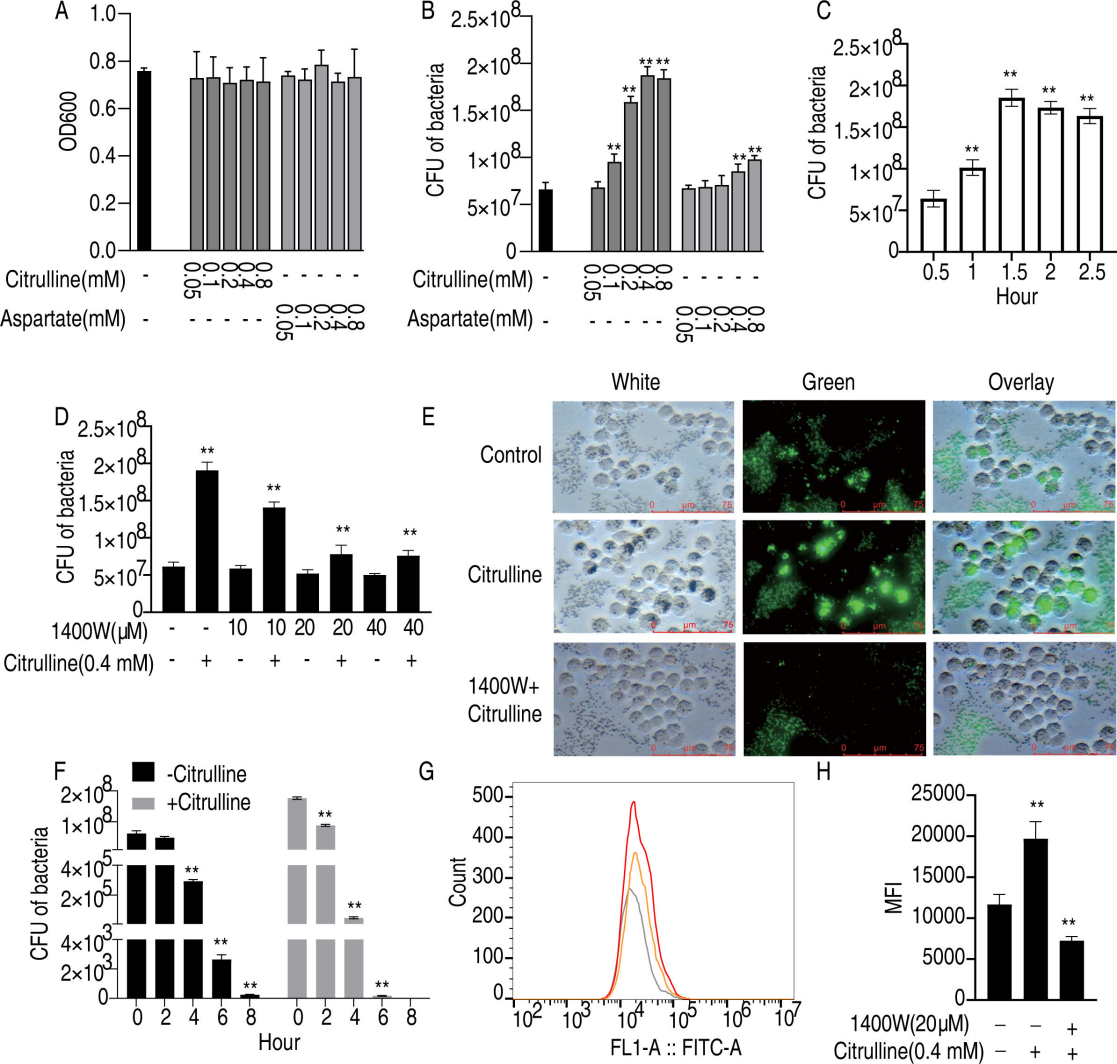
Effect of citrulline and aspartate on phagocytosis activity in HD11 macrophages. (**A**) Effect of citrulline and aspartate on *Salmonella Enteritidis* growth. (**B**) Effect of different concentrations of citrulline and aspartate on the ability of HD11 to phagocytose *Salmonella Enteritidis*. (**C**) Evaluation of phagocytosis time on the ability of HD11 to phagocytose *Salmonella Enteritidis* for citrulline. (**D**) Effect of 1400W on the citrulline-induced phagocytosis activity. (**E**) Effect of citrulline on the level of intracellular *Salmonella Enteritidis* in HD11 macrophage. (**F**) Fluorescence microscopy photographs of citrulline-induced phagocytosis of fluorescent microspheres. (**G and H**) Flow cytometric measurement of citrulline-induced phagocytosis of fluorescent microspheres. **P < 0.01.

Given that citrulline and aspartic acid are common substances identified in Fig. 2D and 3D, they were selected as candidate target substances. Previous studies have shown that NO and the urea cycle in the arginine synthase participate in macrophage activation, influencing the phagocytic activity of phagocytes and thereby contributing to the immune response against infections (20–22). Consequently, this experiment utilized the chicken macrophage cell line HD11 for *in vitro* functional studies. Initially, different concentrations of citrulline and aspartic acid were added to the bone marrow-derived macrophages complete medium, and changes in bacterial growth rates were assessed with or without metabolites. The results indicated that exogenous citrulline and aspartic acid did not affect bacterial growth (Fig. 6A). Subsequently, the effects of exogenous citrulline and aspartic acid on the phagocytosis of *Salmonella Enteritidis* by HD11 cells were examined. Both substances enhanced macrophage phagocytic capacity, with citrulline demonstrating the most significant effect, particularly at a concentration of 0.4 mM (Fig. 6B). Therefore, 0.4 mM citrulline was selected for subsequent studies. The optimal time for citrulline to promote phagocytosis was determined to be 1.5 h (Fig. 6C). To ascertain whether citrulline enhances phagocytosis by improving the urea cycle and increasing NO content, the iNOS inhibitor 1400W was added. The results showed a dose-dependent decrease in citrulline’s phagocytic ability with increasing concentrations of 1400W (Fig. 6D). To directly assess the effects of citrulline and the urea cycle on macrophage phagocytosis, 10 µM FITC fluorescent microspheres were used in place of *Salmonella Enteritidis*. Cells were imaged under a fluorescence microscope after engulfing fluorescent microspheres. The results showed that the addition of citrulline increased the content of fluorescent microspheres in the cells, while 1400W blocked this effect (Fig. 6E). Flow cytometry results corroborated that citrulline increased fluorescence intensity in macrophages, with 1400W reducing citrulline’s phagocytic enhancement (Fig. 6G and H). Lastly, the ability of citrulline to clear intracellular bacteria post-phagocytosis was evaluated. While citrulline initially increased the intracellular bacterial content, the final clearance time was higher than that of the control group (Fig. 6F). These findings suggest that citrulline enhances macrophage phagocytic ability through a mechanism involving NO promotion.

### Citrulline enhances bacterial clearance and survival in chickens infected with *Salmonella Enteritidis*

**Fig 7:**
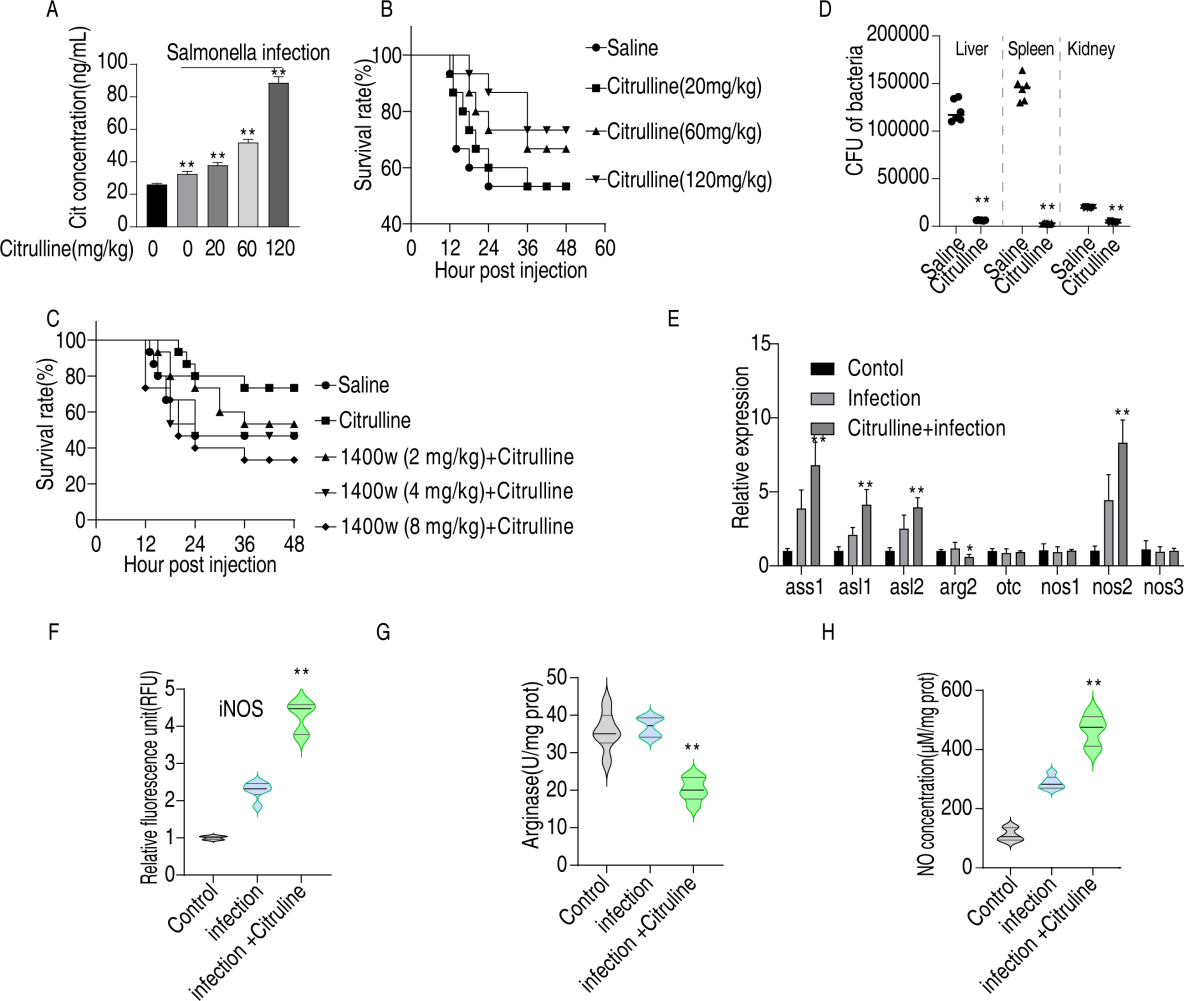
Validation of the biological function of citrulline in *vivo*. (**A**) Effect of citrulline supplementation on the concentration of citrulline in chicken serum. (**B**) Effect of citrulline supplementation on survival rate against *Salmonella Enteritidis* infection. (**C**) Effect of 1400W on citrulline-promoted survival rate against *Salmonella Enteritidis* infection. (**D**) Effect of citrulline on clearance of bacteria in the liver, spleen, and kidney. (**E**) Transcript levels of urea cycle-related genes in citrulline-supplemented chicken following *Salmonella Enteritidis* infection. (**F**) iNOS activity, (**G**) arginase activity, (**H**) NO concentration level in citrulline-supplemented chicken following *Salmonella Enteritidis* infection. *P < 0.05, **P < 0.01.

To investigate the *in vivo* effects of citrulline and the urea cycle in chickens infected with *Salmonella Enteritidis*, chickens were administered 20, 80, and 120 mg of citrulline orally for three consecutive days, followed by a challenge with 3 × 10^9^ CFU of *Salmonella Enteritidis*. Plasma citrulline concentrations were first measured in each experimental group (Fig. 7A). Exogenous citrulline administration significantly elevated plasma methionine levels; concentrations in the infected group were higher than in uninfected controls but remained lower than those in the citrulline-supplemented group. The mortality rate was statistically analyzed. The results indicated that the survival rate of chickens increased significantly with higher doses of citrulline, with the maximum survival rate improving by 20% (Fig. 7B). Additionally, the impact of 1400W on citrulline efficacy was examined, revealing that the presence of 1400W inhibited the effects of citrulline, and at a high concentration (4 mg/kg), 1400W resulted in a higher mortality rate than the control group (Fig. 7C). Furthermore, bacterial clearance rates in the chicken viscera were assessed, showing that citrulline gavage significantly enhanced bacterial clearance efficiency in the spleen, liver, and kidney compared to the non-treated group (Fig. 7D). Lastly, transcriptional expression levels of urea cycle-related genes, iNOS, arginase activity, and NO content were measured in the spleen of the citrulline-treated group. The findings demonstrated that citrulline increased the expression of genes associated with NO production and elevated NO content in chickens while inhibiting arginase activity, consistent with in *vivo* cell experiments (Fig. 7E through G). These results suggest that citrulline promotes NO accumulation, enhances bacterial clearance efficiency, and improves survival rates following *Salmonella Enteritidis* infection by upregulating genes related to NO production.

**Fig 8:**
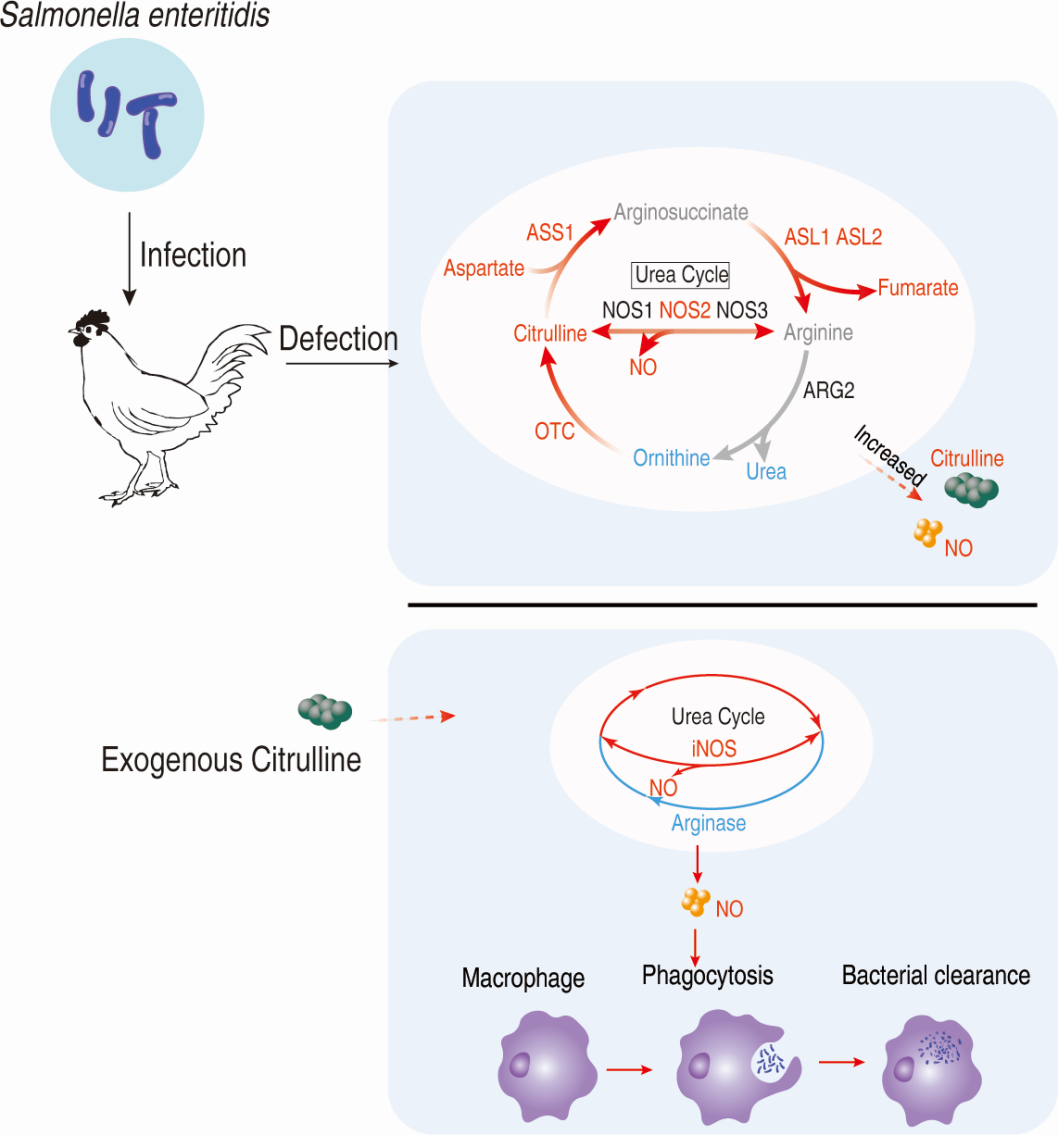
Mechanistic diagram.

A model demonstrating the proposed mechanism is presented in Fig. 8.

## DISCUSSION

*Salmonella Enteritidis* is a prevalent issue in the poultry industry worldwide. On one hand, *Salmonella Enteritidis* infection causes poultry diseases, negatively impacts economic benefits, and threatens the sustainable development of the farming industry. On the other hand, as a zoonotic pathogen, *Salmonella Enteritidis* can be transmitted to humans through poultry products, posing a serious threat to human health and safety (23). Antibiotic treatment has been an effective means of preventing and treating *Salmonella Enteritidis*; however, irrational use of antibiotics has led to the emergence of drug-resistant bacteria and antibiotic residues, exacerbating the challenges in its prevention and treatment (24, 25). Therefore, exploring new strategies for bacterial prevention and treatment is imperative.

In this study, we determined the metabolome of chickens infected with *Salmonella Enteritidis* and found that post-infection levels of citrulline, aspartic acid, and fumaric acid increased with higher infection concentrations. It was speculated that these three metabolites might be associated with the chickens’ resistance to bacterial infection. Differential metabolites were further enriched and analyzed using OPLS-DA. Notably, citrulline and aspartic acid emerged as candidate target substances, and the arginine synthesis metabolic pathway, involving these two metabolites, was among the enriched pathways, ranking second. Based on these findings and functional experiments, citrulline was identified as a biomarker for anti-infection research. In *vivo* and in *vitro* experiments revealed that exogenous citrulline enhances the phagocytic ability of macrophages, improves the clearance of *Salmonella Enteritidis* in chickens, and increases their survival rate post-infection. In this study, we administered citrulline at doses up to 120  mg/kg and observed a maximal protection rate of 20%, which is insufficient for complete therapeutic efficacy. Future investigations will explore higher citrulline doses to establish its dosing ceiling and identify any inherent limitations. Notably, Zhao et al. demonstrated that while glutamine alone modestly improved survival in bacterially infected mice, co-administration with ampicillin achieved 100% protection (26). This finding underscores the potential of combining citrulline with specific antibiotics to attain full therapeutic benefit. Pursuing such synergistic strategies represents a key objective for our subsequent research. Collectively, these results highlight that metabolic modulation can significantly augment host antibacterial defenses.

Innate immunity is the body’s first line of defense against bacterial infections, relying on a range of cellular and molecular mechanisms to recognize and eliminate pathogens (27). At the forefront of innate immune responses are phagocytic cells, including macrophages, which play critical roles in detecting, engulfing, and destroying bacterial invaders (28, 29). Recent research has uncovered that metabolites play a pivotal role in regulating the antibacterial functions of phagocytic cells, marking a significant advancement in the field of immune metabolism. Metabolites are not just byproducts of cellular processes but also act as critical signaling molecules that can influence the immune response. One of the key findings in this area is the impact of metabolites derived from the tricarboxylic acid cycle, such as succinate and fumarate, on phagocyte function. For instance, succinate accumulation has been shown to enhance the production of reactive oxygen species, boosting the bactericidal activity of macrophages (30). Similarly, fumarate can modify proteins through succinylation, affecting various signaling pathways involved in the immune response (31). Additionally, metabolites, such as kynurenine and its derivatives, can modulate the inflammatory response and affect the function of phagocytes, influencing their capacity to clear bacterial infections (32–34). Our study found that citrulline enhances the phagocytic ability of chicken phagocytes HD11 toward *Salmonella Enteritidis* and fluorescent microspheres. Additionally, citrulline was observed to improve the bacterial clearance capacity of HD11 cells post-phagocytosis, although the underlying mechanism remains unknown. These findings suggest that metabolic regulation of phagocytes may offer a novel approach to combating bacterial infections.

Citrulline is a non-essential amino acid that plays a critical role in the urea cycle. Citrulline is synthesized from ornithine and can be converted into arginine. The urea cycle is a crucial metabolic pathway responsible for converting toxic ammonia into urea for excretion. Beyond its primary role in nitrogen detoxification, emerging research has uncovered its significant influence on immune function and the body’s defense against bacterial infections. The intermediate product NO, a critical effector molecule in macrophage-mediated bacterial clearance (35, 36). NO possesses potent antimicrobial properties, enabling macrophages and other immune cells to effectively combat bacterial pathogens (37–39). Our study found that exogenous citrulline supplementation increases the expression of genes related to the citrulline-arginine-NO pathway, while having no effect on the arginase gene of the arginine-ornithine pathway in *vitro* cell experiments, though it inhibits arginase expression in *vivo*. These findings are consistent with those of Victoria A. Uyanga et al. (40), who reported that dietary L-citrulline supplementation modulates nitric oxide synthesis and antioxidant status in laying hens under heat stress (40). The key core enzyme nitric oxide synthase, arginase activity, and nitric oxide content detection results further confirmed these findings. The addition of the nitric oxide synthase inhibitor 1400W blocked citrulline’s ability to promote phagocytosis and improve survival rates post-infection, indicating that citrulline enhances the ability of chickens to resist Salmonella infection by increasing NO production within the urea cycle. Regarding arginase, previous studies have shown that elevated arginase activity can lead to decreased NO levels, potentially impairing the ability of macrophages and other immune cells to kill bacteria, which supports our findings (41–43). Thus, these results indicate that enhancing nitric oxide production is an effective mechanism by which citrulline exerts its anti-Salmonella infection effects.

In conclusion, we measured the metabolome of chickens infected with *Salmonella Enteritidis* and found significant metabolic changes corresponding to the infection intensity. The identified biomarker, citrulline, improved the clearance of *Salmonella* from chicken internal organs and enhanced the survival rate of chicken post-infection. This function is achieved by enhancing the urea cycle to produce nitric oxide. Our study provides a novel approach for the prevention and control of *Salmonella Enteritidis* infection in chickens.

## MATERIALS AND METHODS

### Bacterial strains and culture conditions

*Salmonella enterica* CMCC 50041 was stored in our laboratory. A single colony was propagated overnight for 16 h at 37°C in Luria-Bertani (LB) broth. The cultures were then diluted 1:100 with fresh LB medium and incubated at 37°C for 6 h. The bacteria were subsequently collected, washed three times with saline, resuspended in saline, and adjusted to an OD600 of 1.0 for use in virulence assays.

### Metabolomic profiling

A non-targeted metabolomics approach was used for chicken spleen analysis. Briefly, 25 mg of spleen samples were weighed and cut into 1 mL extraction solution (methanol:water = 4:1). The solution was vortexed for 30 s, sonicated for 10 min at 35% intensity with 2 s intervals and 3 s rest periods, then left to stand for 30 min. After centrifugation at 12,000 rpm for 10 min at 4°C, the supernatant was collected and transferred to a new tube. The extract was dried using a LamConco vacuum concentrator. Next, 80 µL pyridine with 20 mg/mL methoxyamine hydrochloride was added for methoximation, and the mixture was heated at 37°C for 3 h. Then, 80 µL of N-methyl-N-trimethylsilyltrifluoroacetamide was added and shaken at 37°C for 30 min. Differential metabolites were detected using an Agilent 7890A GC and an Agilent 5975C inert XL mass selector (Agilent Technologies).

### GC-MS analysis

Initial peak detection and mass spectral deconvolution were conducted using Agilent 6.0 software. Identified metabolites from GC-MS were cross-referenced with the National Institute of Standards and Technology (NIST 08) Mass Spectral Library, and duplicate metabolites were consolidated. Data normalization was performed using the concentrations of internal standards and total intensity. The resulting normalized peak intensities from each file constituted a single RT-m/z matrix, serving as the basis for all subsequent statistical analyses. Hierarchical clustering was executed with the gplots package in R. The Z-score was utilized to analyze the normalized areas of differential metabolites. Multivariate statistical analyses, including PCA and S-plot analysis, were performed using SIMCA-P + 12.0.1 software (Umetrics, Umea, Sweden). Hotelling’s T2 (95%) was applied to identify strong outliers, depicted as cycles (ellipses) in the figure; values within the ellipse are considered normal, while those outside are deemed abnormal. Metabolic pathway enrichment was carried out using the MetabolAnalyst computational platform (https://www.metaboanalyst.ca/).

### Measurement of inducible nitric oxide synthase, arginase, and NO

Chicken spleen samples were collected and added to PBS, then finely cut with scissors. The samples were subjected to ultrasonic disruption on ice, followed by centrifugation at 12,000 rpm for 10 min at 4°C to remove insoluble material. The supernatant was collected, and the protein concentration was determined using a BCA kit (P0009; Beyotime Institute of Biotechnology). The total protein concentration of the supernatant was adjusted to 200 µg. Each sample was assayed in a 96-well microplate format according to the manufacturer’s instructions. NO and iNOS levels were detected using double antibody sandwich enzyme-linked immunosorbent assay kits from Beyotime (S0023, S0025). Arginase activity was measured using the Arginase Activity Assay Kit from Solarbio Life Sciences (BC5555).

### RNA isolation and RT-qPCR

Total RNA was extracted using the TRIZOL method (TRIzol, THERMO 15596026), followed by phase separation with chloroform, precipitation with isopropyl alcohol, washing with 75% ethanol, and re-dissolution in DEPC-treated water. One microgram of RNA was reverse-transcribed using a kit from Accurate Biotechnology (AG11728, ACCURATE BIOTECHNOLOGY [HUNAN] CO., LTD, Changsha, China). Quantitative reverse transcription real-time polymerase chain reaction (qRT-PCR) was performed using a SYBR Green Premix qPCR Kit (AG11701, ACCURATE BIOTECHNOLOGY [HUNAN] CO., LTD, Changsha, China) in 384-well plates. All samples were run on a LightCycler 480 system (Roche, Germany), with four independent samples assayed for both control and test groups. GAPDH was used as an internal control to normalize the relative expression levels of the target genes, the primers were listed in the supplementary information Table S1.

### Cell culture and phagocytosis assay

Chicken macrophage HD11 cells were cultured in DMEM (HyClone) containing 10% FBS and 1% chicken serum at 37°C with 5% CO_2_. Phagocytosis by macrophages was examined as previously described. Briefly, HD11 cells were harvested using CaCl_2_- and MgCl_2_-free PBS containing 5 mM EDTA and plated at 5 × 10^6^ cells/well in 6-well plates. For experiments involving citrulline or inhibitors at specified concentrations, cells were serum-starved overnight and then incubated with the indicated molecules in serum-starved medium (DMEM/0.5% serum) for the specified time.

After 6 h of pretreatment, fluorescent microbeads (FluoSpheres carboxylate-modified microspheres, 1.0 µm, yellow-green fluorescent; Beijing Zhongkeleiming Daojin Technology Co., Ltd.) and *S. enterica* CMCC 50041 at a multiplicity of infection of 100 were used for a 1.5 h phagocytosis assay. Phagocytosis was halted by vigorously washing the macrophages with cold PBS. The cells were washed at least four times with cold PBS. For the fluorescent microbead group, cells were processed for fluorescence microscopy and FACS analysis. For the bacterial group, cells were enumerated using the standard pour plate method with serial dilutions.

### Statistical analysis

Data are presented as means ± SEM. Comparisons between two groups were performed using an unpaired *t*-test (Prism 5.0; GraphPad Software, San Diego, CA, USA) for data with a Gaussian distribution and equal variance, or with Welch’s correction if variances were unequal. For non-Gaussian data, the Mann-Whitney U test (Prism 6.0; GraphPad Software) was applied. Comparisons among more than two groups were conducted using one-way analysis of variance, followed by Dunnett’s multiple comparisons test (Prism 5.0; GraphPad Software) for Gaussian data with equal variance, or the Kruskal-Wallis test with Dunn’s post hoc test for non-Gaussian data. Gaussian distribution was assessed using the D’Agostino-Pearson omnibus and Kolmogorov-Smirnov tests (Prism 5.0; GraphPad Software). Variance homogeneity was evaluated with the homogeneity of variance test (SPSS 22.0) or the Brown-Forsythe test (Prism 6.0; GraphPad Software). Detailed statistical methods and significance levels (*P < 0.05, **P < 0.01) are provided in the figure legends.

## SUPPLEMENTAL MATERIAL

Supplemental material spectrum.01546-25-s0001.docx Figure S1 and Table S1.

ASM does not own the copyrights to Supplemental Material that may be linked to, or accessed through, an article. The authors have granted ASM a non-exclusive, world-wide license to publish the Supplemental Material files. Please contact the corresponding author directly for reuse.
