# Supplementary material for: Citrulline-enhanced chicken resistance to Salmonella Enteritidis infection via urea cycle modulation and nitric oxide production
Source: Microbiol Spectr. 2025 Nov 11;13(12):e01546-25. doi: 10.1128/spectrum.01546-25 (PMC12671226; doi:10.1128/spectrum.01546-25)
Supplement: Supplemental material — Figure S1 and Table S1. [file spectrum.01546-25-s0001.docx]

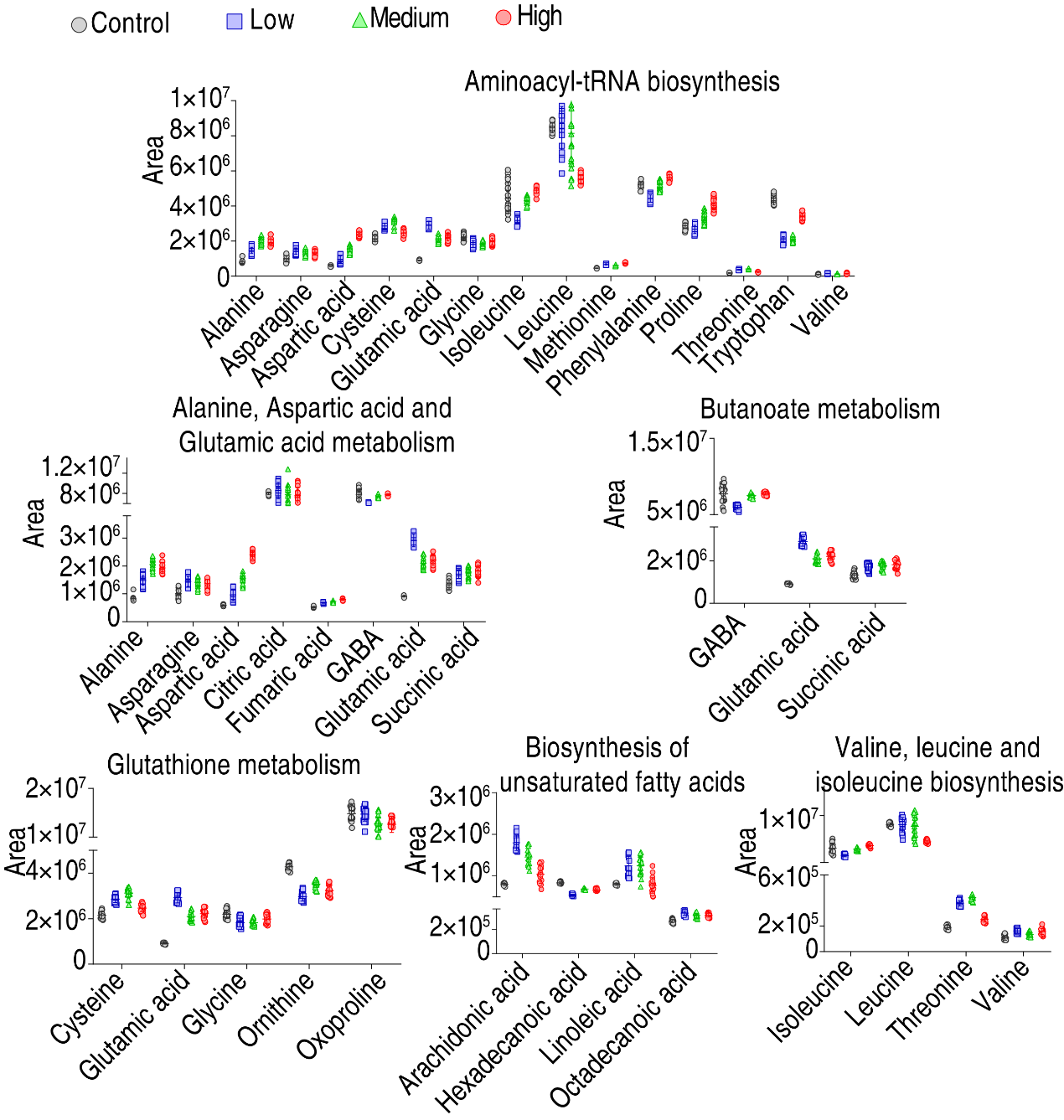


Figure S1 Abundance of metabolites of each pathway

| Table S1 Primers for qpcr | |
| --- | --- |
| Gene name | Primer(5'---3') |
| ARG2-F | agttcccaatgatgaactgtac |
| ARG2-R | aagtgccaagctgtgatcac |
| NOS3-F | cgggacttcatcgaccaata |
| NOS3-R | gcctcctgcagctgatatgt |
| NOS2-F | tgatgccaaattacacagtc |
| NOS2-R | tggtccgacaattgataacc |
| NOS1-F | gcacccagagctgttgagtt |
| NOS1-R | ccttgagaagctcgttgtcttc |
| ASL1-F | tggcctggaaaagatctctg |
| ASL1-R | cttcaggagcagtttcagatca |
| ASL2-F | tccattgcctgtgatcagag |
| ASL2-R | ttcattggcagtgtgaatatcc |
| ASS1-F | gggatgaagtccagaggtat |
| ASS1-R | tgcttcaggaactcacactc |
| OTC-F | ggccaccaaatcaaatgaat |
| OTC-R | cccaggtgaatgtcttgttt |
| ACY1-F | cgagaagttgcacaaagtga |
| ACY1-R | caccacgttgaaggagactc |
| CPS1-F | gggaacccggttaaagttgt |
| CPS1-R | aataatgagcccatcatactcc |
| GLUD1-F | gacgaccccaacttcttcaa |
| GLUD1-R | tgctaaggctttcacttcatc |
